# Microscale Electrical Resistivity Measurements to Investigate Particle Distribution

**DOI:** 10.1021/acs.langmuir.4c03429

**Published:** 2025-01-07

**Authors:** Emre Baburoglu, Maureen H. Tang, Nicolas J. Alvarez

**Affiliations:** † Materials Science and Engineering, Drexel University, 3141 Chestnut Street, Philadelphia, Pennsylvania 19104, United States; ‡ Chemical and Biological Engineering, Drexel University, 3141 Chestnut Street, Philadelphia, Pennsylvania 19104, United States

## Abstract

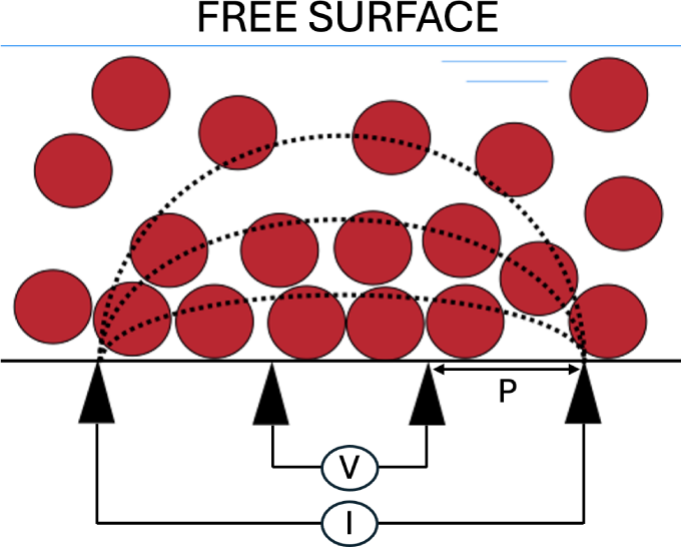

The functional performance of a particulate thin film depends greatly on the particle distribution that forms during drying. In situ methods for monitoring the impact of different processing parameters on the distribution of particles currently require expensive and specialized equipment. This work addresses this gap by miniaturizing a geophysical prospecting method to thin-film applications. In this method, four-electrode resistivity measurements at variable probe spacing detect changes in the vertical particle concentration profile. A heuristic colloidal drying model describes the particle distribution during drying in terms of the relative effects of Brownian diffusion, sedimentation, and evaporation. For sedimentation- and evaporation-dominated drying, the film is modeled as two stratified layers of different concentrations. Solving this model simultaneously alongside Laplace’s equation for electrostatic resistance identifies the parameters necessary to distinguish between diffusion-, sedimentation-, and evaporation-dominated drying. For resistive particles in a conductive solvent, simulations predict that the normalized thickness of the top layer, δ_*t*_/*H*_0_, must exceed a critical value to distinguish between different drying regimes. The heuristic model results are validated theoretically by comparison to a physics-based drying model. Model predictions are experimentally validated by fabricating a custom microlithography four-line probe device and measuring the transient resistance of systems for which the drying mechanism is known. This work offers a low-cost and in situ method to identify drying mechanisms and extract physical parameters that better characterize the processing-structure–function relationships for many coatings.

## Introduction

Particulate thin films are used in a diverse number of technologies ranging from paints to energy storage.^1,2^ As these films are often much larger laterally than they are vertically, the vertical distribution of particles significantly impacts performance. In particular, the increasing demand for electric vehicles motivates study of particle distribution effects on battery electrode performance. Yari et al. demonstrated these effects and found that a higher concentration of carbon black particles near the vicinity of the current collector improved the performance of a lithium-ion battery cathode.^3^ Conversely, other studies have found that a homogeneous distribution of carbon black particles (i.e., a lack of vertical gradients) is needed to optimize electrode performance.^4,5^ Another example is demonstrated by Jaiser et al. where the migration of polymer binder to the film surface during drying is detrimental to the performance of the lithium-ion battery cathode.^6^

**Figure 1:**
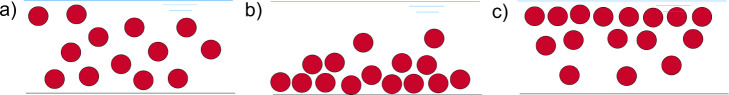
Illustrations of the three drying regimes. (a) Diffusion dominated drying, (b) sedimentation dominated drying and (c) evaporation dominated drying.

The particle distribution develops during drying and therefore hinges on the processing conditions that dictate the drying mechanism.^7−9^ As a particulate coating dries, the distribution of particles normal to the substrate depends on the interplay between evaporation, sedimentation and diffusion. When evaporation dominates drying, the height of the air–liquid interface decreases faster than the particles can diffuse, forming a particle dominated skin layer (often called the “consolidation front”). This mechanism is illustrated in Figure 1c. When sedimentation dominates drying, particles fall to the bottom of the film faster than the film dries (Figure 1b). Lastly, diffusion dominated drying ensures that thermal Brownian diffusion nullifies any concentration gradients developed by drying or sedimentation (Figure 1a).^7^

Although the physics of these different regimes are well understood, it is difficult to quantify the drying mechanisms experimentally. Current methods for investigating microstructural changes in colloidal thin films are time-consuming, difficult to perform *in situ*, and unsuitable for large-scale applications. Many involve large-scale specialized equipment. For example, the most common methods are cryogenic scanning electron microscopy (Cryo-SEM),^10−15^ X-ray scattering,^16−19^ and magnetic resonance imaging (MRI).^20−22^

Electrical measurements offer a much cheaper and faster alternative and have been used to study colloidal microstructure in various ways. For example, Narayanan et al. correlated the through-plane resistance to microstructure of a carbon black and ethylene carbonate slurry under varying shear stress.^23^ Liu et al. correlated the low-frequency dielectric strength of a carbon black suspension containing polyvinlyidene difluoride in *N*-methyl-2-pyrrolidone to its fluid Mason number and thus aggregate size.^24^ Bai et al. used impedance measurements with equivalent circuit modeling to characterize the network structure of a lithium ion battery cathode slurry.^25^ However, such two-electrode measurements can be convoluted by interfacial effects at the electrode with the bulk resistivity to be characterized.

A four-electrode measurement is an in-plane resistivity measurement where current passes between outer working electrodes and potential is measured at the inner sensing electrodes. Measuring voltage at the inner sensing electrodes rather than the outer working electrodes is experimentally necessary to avoid interfacial processes from contributing to the measured resistance. This method, which has been used for groundwater exploration in geophysical prospecting for nearly a century, derives from the fact that the electric field penetrates deeper into the subsurface with greater spacing between electrodes. By altering the probe spacing, one can measure the apparent resistivity of the ground at different depths, allowing the collection of accurate information about the location and concentration of groundwater.^26^ This particular property of four-electrode measurements has since been applied to deconvolute electronic conductivity from contact resistance in lithium-ion battery electrodes by Lanterman et al,.^27^ Similarly, Shiraki et al. characterized the vertical resistance profile of a Si(111) crystal,^28^ and Clarysse et al. profiled the carrier depth of Si and Ge sheets.^29^

Herein, we miniaturize this geophysical prospecting method to study the particle distribution in particulate films during drying. If the particles or the surrounding medium are electrically conductive, the vertical concentration gradients that form during drying (described above) will correspond to vertical resistivity gradients. Therefore, if the thin film is coated on a substrate embedded with multiple electrode arrays, the apparent resistivity measured will depend on the spacing between the adjacent electrodes of each array. In this study, we develop a heuristic model to investigate the parameters necessary to distinguish between the drying regimes, validate the heuristic model against an accepted physics-based drying model, and build a four-electrode device to confirm the modeling results experimentally.

## Model Development

### Four-Point Probe Electrode Model

**Figure 2:**
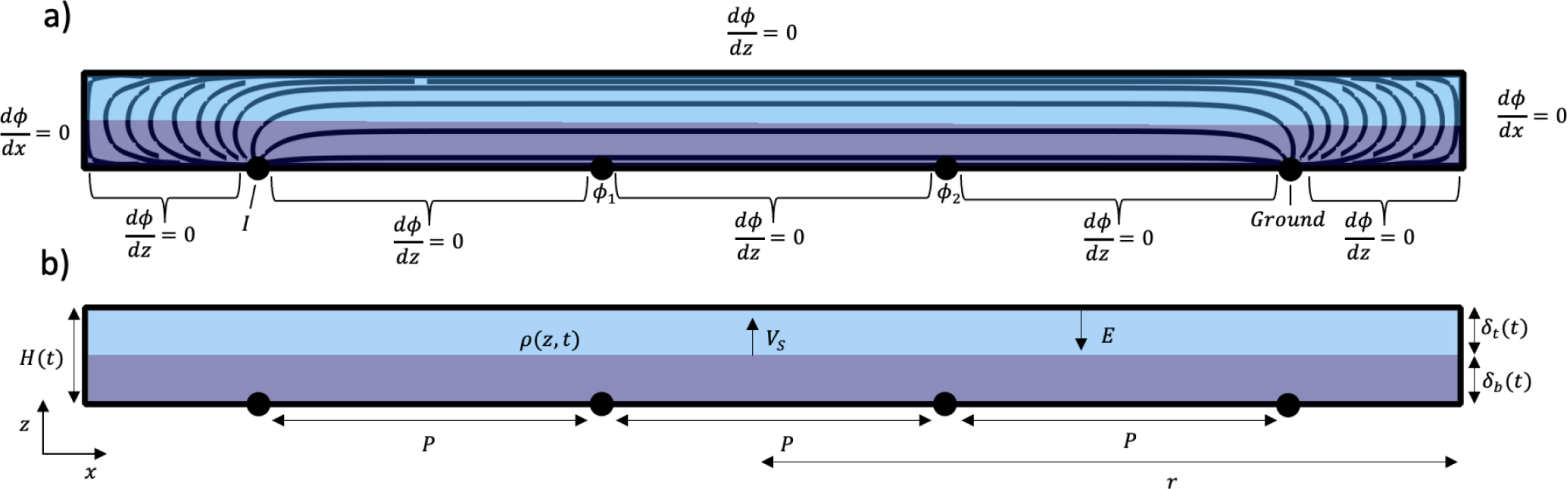
Schematic of the four-electrode measurement simulation on a two-layered sample modeling a drying particulate film. (a) Describes the boundary conditions applied when solving Laplace’s equation in terms of the electric potential ϕ. The solid lines represent the electric field lines produced by COMSOL. (b) Describes the film dimensions and drying parameters.

Figure 2 illustrates a vertical cross section of the geometry used in this study. Four infinitesimally small colinear point probe electrodes with equidistant spacing, *P*, on a cylindrical substrate with radius *r*, contact a sample of thickness, *H*. Note that *r* ≫ *P*. The electric field is governed by Laplace’s equation, i.e.,∇^2^*ϕ* = 0, for electric potential ϕ. All the boundaries are insulating except for the outer two electrodes, where a current I is applied at the far left electrode, and the far right electrode is designated as ground. ϕ is determined at the inner two electrodes, which yields the sample resistance *R* = Δ*ϕ*/*I*. The resulting *R* is then converted to resistivity ρ by multiplying by a shape factor *S*. Assuming equidistant probe spacing, isotropic electronic conductivity, and *r* ≫ *P*, *S* = *πH*/ ln(2) when *H* ≪ *P* and *S* = 2π*P* when *H* ≫ *P*.^26^ Simulations are performed at two different values of *P*, 10 mm and 200 mm, to simulate *P*/*H*_0_ > 2 and *P*/δ_b_(*max*) < 0.5, where *H*_0_ = 100 mm is the initial film thickness, and δ_b_(*max*) is the maximum bottom layer thickness.

### Heuristic Colloidal Drying Model

As a colloidal film dries, the measured resistance is a function of morphological changes that come about from (i) solvent evaporation and (ii) changes and gradients in particle concentration. These effects can be simulated via a simple drying model and a relationship between resistivity and particle concentration. In this work, we assume that vertical gradients in concentration can be represented by the changes in thickness and resistivity of two distinct stratified layers. The transient thicknesses of the bottom layer and top layer are represented by δ_b_(*t*) and δ_t_(*t*), respectively. The changes in concentration lead to a variation of resistivity, ρ(*z*, *t*), that is represented by the following S-type function:
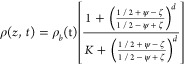
1where *ζ* = *z*/*H*_*0*_ is the vertical coordinate scaled by the initial film thickness, *ψ* = δ_*b*_(*t*)/*H*(*t*), i.e., the bottom layer thickness scaled by the film thickness, the exponent *d* defines the sharpness of the S-curve, and *K* = *ρ*_b_(*t*)/*ρ*_t_(*t*), where *ρ_b_*(*t*) and *ρ_t_*(*t*) are the resistivities evaluated at *z* = 0 and z = *H*(*t*), respectively. The functional form of ρ_*b*_(*t*) and ρ_*t*_(*t*) depends on the drying regime. eq 1 is a continuous function that allows the film to be defined as a single layer with properties that depend on height and time. The parameter *d* sets the slope of the interface between the top and bottom layers. For *d* > 2, the interface between top and bottom layers is sharp, and thus, the output resistivity is a weak function of *d*, see Figures S3 and S4. In this study, we assume *d* = 50 and δ_*b*_/*H*_0_ > 0.2.

For this work, we assume that the drying leads to a linear decrease in height given by,
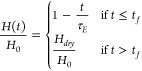
2where *H*_*dry*_ is the final dried film thickness, *t*_*f*_ is the final drying time, and τ_*E*_ = *H*_0_/*E* is the evaporation time constant and *E* = (*H*_0_ – *H*_*dry*_)/*t*_*f*_ is the evaporation rate, given in terms of the velocity of the air–liquid interface.

#### Diffusion Dominated Drying

**Figure 3:**
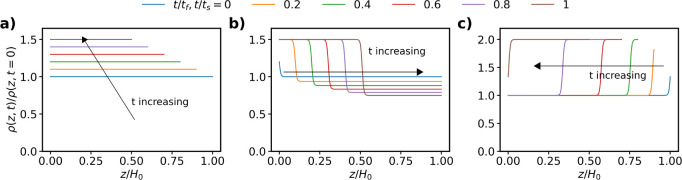
S-curves representing the vertical resistivity profile of the colloidal thin film when drying is (a) diffusion dominated (τ_*E*_ = 100 s, *t*_*f*_ = 50 s, *H*_*dry*_ = 50 mm), (b) sedimentation dominated (τ_*S*_ = 100 s, *t*_*S*_ = 50 s, *H*_*sed*_ = 50 mm) and c) evaporation dominated (τ_*E*_ = 100 s, *t*_*f*_ = 50 s, *H*_*dry*_ = 50 mm).

When drying is diffusion dominated, the fast rate of diffusion normalizes any gradients in concentration such that the film dries homogeneously, i.e., ρ(*z*, *t*) = ρ_*b*_(*t*). Thus, the only changes in resistivity are due to the decrease in film height (volume of solvent) and thus the increase in solute concentration. If the particles are resistive while the solvent is conductive, the resistivity in the drying film is given by a piecewise function:
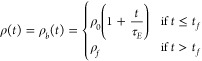
3where ρ_0_ is the initial resistivity of the wet film, ρ_*f*_ is the resistivity of the dried film, and τ_*E*_ is the same evaporation time constant shown in eq 2. Combining eqs 1 and 3 yields the transient and spatial dependence of resistivity as a colloidal film dries homogeneously. Figure 3a shows the normalized resistivity through the vertical position of the film at different normalized times.

#### Sedimentation Dominated Drying

When drying is sedimentation dominated, particles concentrate in the bottom layer (i.e., the sediment), and the top layer (i.e., the supernatant) dilutes over time. Since sedimentation occurs much faster than evaporation, the change in height of the film is irrelevant and is considered constant with time, i.e., eq 2 becomes *H*(*t*) = *H*_0_. For the case of resistive particles in a conductive solvent, *ρ*_*t*_(*t*) decreases such that ρ_*t*_(*t*) < ρ_*b*_(*t*). In this case, we assume that δ_*b*_(*t* = 0) = 0 and δ_*t*_(*t* = 0) = *H*_0_. For simplicity, the top layer thickness is assumed to decrease linearly with time and is given by
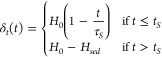
4where time constant for sedimentation is *τ*_*s*_ = *H*_0_/*V*_*S*_, *V*_*S*_ is the sediment layer growth velocity, the final sedimentation time *t*_*S*_ = *H*_*sed*_/*V*_*S*_, and *H*_*sed*_ is the final sediment layer thickness. Assuming a linear dependence of resistivity in time, *ρ*_*t*_(*t*) is given by
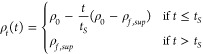
5Where *ρ*_*f*,*sup*_ is the final resistivity of the supernatant. Combining eqs 1, 4 and 5 gives the variation in resistivity for sedimentation dominated drying. Figure 3b shows the resistivity through the layer thickness for different normalized times.

#### Evaporation Dominated Drying

When drying is evaporation dominated, the top of the film concentrates first, and the concentrated domain grows with time. To model this phenomenon, both *ρ*_*t*_(*t*) and *ρ*_*b*_(*t*) are equal to a constant, and the position of the top/bottom layer interface changes with time, and δ_*b*_(*t* = 0) = *H*_0_ and δ_*t*_(*t* = 0) = 0. In other words, the thickness of the consolidated top layer, δ_*t*_(*t*), grows as *H*(*t*) decreases, i.e.,
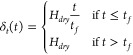
6where *H*_*dry*_ is the final film thickness. Combining eqs 1, 2 and 6 yields the transient and spatial dependence of resistivity under evaporation dominated drying. Figure 3c shows the spatial variation in resistivity for different normalized times for a conductive solvent and resistive solute.

### Physics-Based Drying Model

The heuristic model of eqs 1–6 was selected over a physics-based model based on the ease of moving through parameter space, as will be shown in the Results and Discussion section. We compare the predictions of the heuristic model to a physics-based model from Cardinal et al.^7^ In their model, the vertical concentration profile of the drying colloidal film is determined by solving the following one-dimensional mass conservation equation:
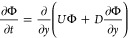
7where Φ is the volume fraction of particles, *y* is the vertical spatial coordinate, and *U* and *D* are the sedimentation velocity and diffusion coefficient, respectively. These are experimentally determined functions of Φ given by,
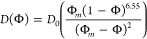
8
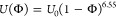
9where *D*_0_ is the Einstein diffusion coefficient, and *U*_0_ is the Stoke’s settling velocity of dilute particles. The equations are solved subject to two boundary conditions: a no-flux boundary condition at the bottom boundary, and an evaporative flux at the air–liquid interface, given by,
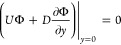
10
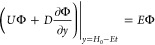
11where *H*_0_ is the initial film thickness, and *E* the evaporation rate. The initial condition is assumed to be the same for all simulations and is given by Φ(*t* = 0, *y*) = 0.4. eqs 7–9 are solved subject to eqs 10 and 11for two values of *U*_0_ = 1 [mm/s] and *U*_0_ = 100 [mm/s] for a fixed *D*_0_ = 100 [mm^2^/s] and *E* = 1 [mm/s]. Note that these parameters correspond to an evaporation Peclet number, Pe = *EH*_0_/*D*_0_ = 1, and a sedimentation number, *N_s_* = *U*_0_/*E* = 1 for the evaporation regime and *N*_*s*_ = 100 for the sedimentation regime. Since the solvent is conductive and particles resistive, the resistivity of the film depends on the concentration (aka volume fraction) of the solvent (1 – Φ). Additionally, as the solvent is assumed to be isotropic, resistivity must be inversely proportional to solvent concentration. Thus, resistivity is represented as a function of 1 – Φ, via:
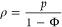
12where *p* is a constant of proportionality.^30^ In this study this value was arbitrarily selected to be *p* = 0.64. The chosen value of *p* has no impact on the results presented in this study.

### Numerical Method

The numerical calculations were performed using COMSOL Multiphysics 5.6. The Electric Currents physics package with the stationary solver was used to simulate the four-point probe measurement. eqs (1^1^–6)6 were parametrized as functions of time and solved analytically. eqs7–11 were solved using the General Form PDE package also using the stationary solver.

### Materials and Experimental Procedures

#### Resistance Measurements

**Figure 4:**
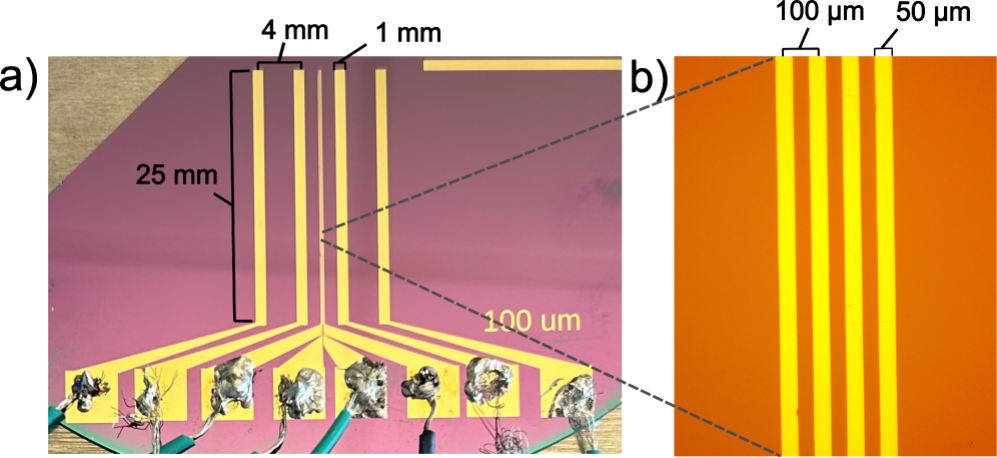
(a) Image of one of fabricated four-line probe devices with center-to-center probe spacing and electrode width of the larger spaced probes labeled (4 mm and 1 mm respectively). (b) 10× magnified image of the smaller spaced probes in the middle of the larger spaced probes with center-to-center probe spacing of 100 μm and electrode width of 50 μm.

Microscopic probes for resistivity measurements were manufactured using standard photolithography procedures in the Singh Center for Nanotechnology. Line electrodes, rather than point electrodes, were chosen based on relative ease of fabrication. *p* values were selected as 4 mm for the larger spaced probes and 40, 50, 100, or 200 μm for the smaller spaced probes. All electrodes were 25 mm in length as shown in Figure 4. To fabricate the electrodes, 1 μm of SiO_2_ layer was first deposited on top of a 4-in. silicon wafer using chemical vapor deposition (Oxford Instruments Plasma Lab 100 PECVD). Next, the wafer was spin coated with a bilayer positive resist consisting of LOR-3A and S1818. The resist was then patterned via direct write (Heidelberg DWL 66+Laser Writer) and developed by soaking in AZ-300 MIF. 100 nm of Au was then deposited with a 3 nm Cr adhesion layer using physical vapor deposition (Kurt J. Lesker PVD 75 PRO-Line E-Beam Evaporator). The excess resist was then stripped by soaking in *N*-methyl pyrrolidone.

**Figure 5:**
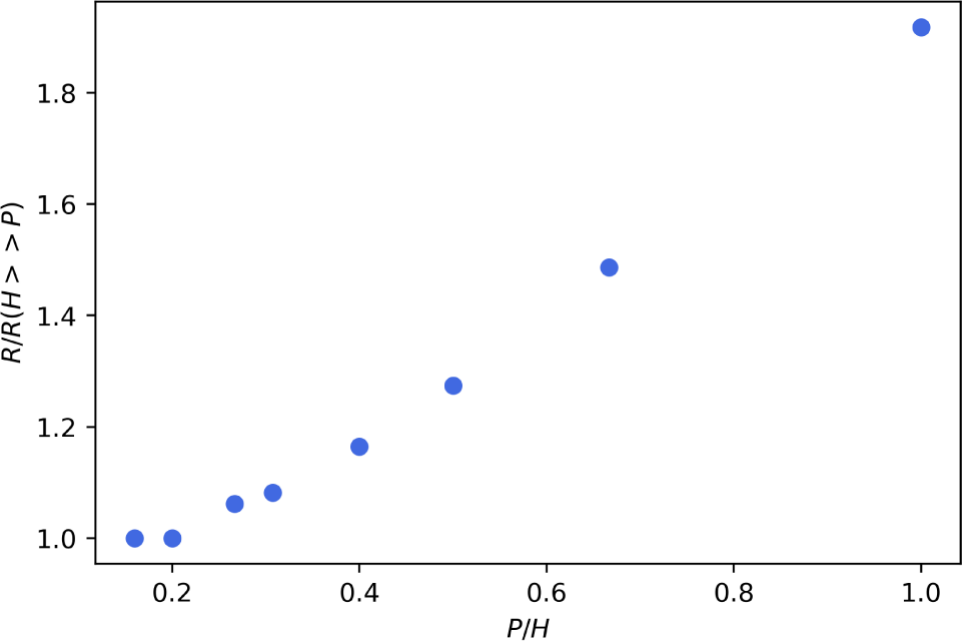
Change in the resistance of a solution of 10 mM Na_2_SO_4_ measured by a probe with *p* = 40 μm normalized by the resistance at large thicknesses with changing P relative to H.

After fabrication, resistance was measured by placing a liquid sample on top of the line probes. The electrode length was controlled by masking the electrodes with Kapton tape. Then, a VMP-3 potentiostat (Biologic USA) applied galvanostatic electrochemical impedance spectroscopy (20 μA, 10 Hz) to measure the resistance. Resistances of the smaller and larger spaced probes were measured in separate experiments to avoid interference between simultaneous electrical signals. The penetration depth of the smaller spaced probe was determined by measuring the resistance of a salt solution (10 mM Na_2_SO_4_) at different thicknesses. The thickness was controlled using the top plate of a parallel plate rheometer covered by a polyoxymethylene (Delrin) part to ensure electrical insulation between the salt solution and the top plate. The bottom plate was the four-line probe device. Figure 5 shows the change in the resistance measured by a smaller spaced probe normalized by the resistance at large thicknesses with changing probe spacing, *P*, relative to the thickness, *H*. At high *P*/*H*, resistance increases with decreasing thickness due to a decrease in the number of current paths available. At low *P*/*H*, resistance is independent of thickness since the electric field cannot penetrate deep enough into the sample to be able to detect the changing thickness. The transition from high to low *P*/*H* occurs at around 0.2 *P*/*H*. This value is a factor of 2.5 lower than the model prediction for idealized point probes. The reason for this discrepancy is likely the finite width and thicknesses of the experimental electrodes in contrast to the infinitesimal simulated electrodes. This experimental parameter is considered when designing the experiments in this study.

#### Proof of Concept Experiments

The transient resistances of samples known to dry homogeneously (diffusion dominated), to form a consolidated skin (evaporation dominated), or to form a sediment (sedimentation dominated) were measured to experimentally validate the model predictions. For the diffusion dominated proof of concept experiment, 550 μL of a solution of 10 mM KCl (Sigma, purity 99%) in water (Milli-Q, resistivity 18.2 MΩ cm) was dispensed on a 6 mm × 14 mm area of the probe covering 6 mm of the length of the line probes and dried. In an ionic solution, ions are able to diffuse much faster than the film or droplet is able to dry. Thus, the drying mechanism will always be diffusion dominated. In their 2019 study, Hipp et al. showed that when a slurry composed of 8 wt % carbon black in propylene carbonate is sheared at a constant shear rate of 100 s^–1^, the slurry sediments.^19^ Thus, for the sedimentation dominated proof of concept experiments, the same carbon black (Vulcan XC-72, Cabot Corporation, bulk density 0.264 g/cm^3^) and propylene carbonate (Sigma-Aldrich, density 1.2 g/cm^3^) slurry formulation was prepared by mixing in a planetary mixer (Thinky Corporation) at 1800 rpm for 7.5 min. A Discovery HR-3 parallel plate rheometer (TA Instruments) was used for this study. The bottom plate of the rheometer was modified to include a four line probe electrode. The slurry was placed on top of the modified bottom plate electrode. The top plate was brought down to the specified measurement height and the excess material was trimmed. The slurry was presheared at 2000 s^–1^ for 1 min followed by a 30 s rest. It was then sheared at 100 s^–1^ to induce sedimentation. Rheological measurements used a 25 mm disposable aluminum top plate covered by a Delrin part to ensure electrical insulation between the carbon black slurry and the top plate. The gap height was set to 1 mm for all experiments presented in this study. The line probes were covered by insulating Kapton tape such that the resistance measurement was limited to a 4 mm gap in the middle of the sample.

In their 2010 study, Cardinal et al. showed that when a suspension consisting of 0.4 volume fraction spherical silica particles in water is dried under drying conditions where Pe ≥ 1 and *N*_*s*_ < 1, with Pe and *N*_*s*_ being the evaporation Peclet number and sedimentation number respectively, a consolidated skin of particles forms at the top of the suspension.^7^ Thus, for the evaporation dominated proof of concept experiments, a similar silica nanoparticle (General Engineering and Research, diameter 200 nm, skeletal density 2.1–2.2 g/cm^3^) suspension, this time suspended in an aqueous solution of 10 mM KCl, was prepared by stirring with a stir bar overnight. 700 μL of the suspension was deposited on an 18 × 18 mm area of the device and dried under conditions of Pe = 77.7 and *N*_*s*_ = 2 × 10^–4^ using a fan. This Pe value was calculated by measuring the drying rate, *E*, using a digital displacement micrometer, as further discussed in Figure S1.

## Results and Discussion

### Numerical Results

**Figure 6:**
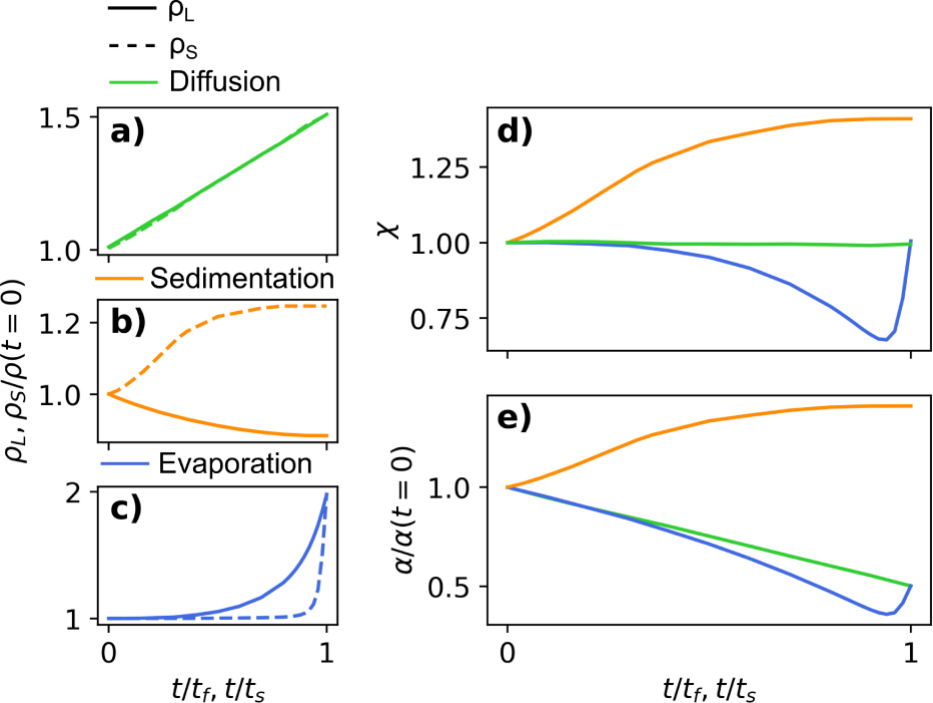
Resistivity observed by the probe with *P*/*H*_0_ = 0.1 (dashed line, ρ_*S*_) and *P*/*H*_0_ = 2 (solid line, ρ_*L*_) when drying is (a) diffusion (), (b) sedimentation (, ), and (c) evaporation (, ) dominated. (d)The resistivities observed by the two probes for the three drying regimes represented in terms of the ratio of the two observed resistivities (χ). (e) The resistances observed by the two probes for the three drying regimes represented in terms of the ratio of the two observed resistances (α).

Figure 6 shows the change in normalized resistivity for two four-point probes with normalized spacing: *P*/*H*_0_ = 0.1 (dashed line) and *P*/*H*_0_ = 2 (solid line). The resistivity observed by the smaller and larger spaced probes are denoted by ρ_*S*_ and ρ_*L*_, respectively. Figure 6a–c represent the result for the three different drying regimes: (a) diffusion dominated, (b) sedimentation dominated, and (c) evaporation dominated. Figure 6a shows the diffusion dominated case, whereby the concentration profile is uniform through the film as a function of time. As expected, both probes give the same increase in resistivity. Therefore, for the diffusion dominated case, we observe a resistivity that is independent of probe spacing. Figure 6b shows the result when drying is sedimentation dominated. Since the resistive particles are depositing near the electrode surface with time, the smaller spaced probe shows a sharp increase in resistivity. The response of the larger spaced probe is more difficult to understand because it depends on the sizes of the sediment layer and supernatant layer (i.e., top layer), which in turn depend on the model parameters. For the values simulated here, the larger spaced probe shows a slight decrease in resistivity because the sediment layer is less resistive than the supernatant layer is conductive. Note that the larger spaced probe would show an increase in resistivity had the consolidated sediment layer been much larger than the supernatant layer. In Figure 6c the physics is evaporation dominated, which means that the concentration of resistive particles is only building up at the air–liquid interface, such that the bulk concentration stays relatively the same. Thus, initially, the two probes see almost no change in resistivity, even though the volume of the film is decreasing. For the simulated case, when *t*/*t*_*f*_ = 0.2 the consolidated layer at the air–liquid interface is large enough to change the resistivity observed by the larger spaced electrode. At later times, the film is thin enough that the smaller spaced electrode also measures the resistive top layer of the film.

The relative measurement of the two probes is useful in observing the different drying regimes. More specifically, the ratio of the two resistivities, denoted as *χ = ρ_S_/ρ*_*L*_, eliminates changes in resistivity due to uniform changes in concentration during drying, such as that observed in Figure 6a. Figure 6d shows χ for all three cases. As expected, the diffusion dominated regime is represented by a horizontal line at χ = 1 for all times. The sedimentation regime is represented by an increase in χ. The evaporation regime is denoted by a minimum in χ, which occurs at a time that depends on the model parameters. Note that the simulation results in Figure 6 are for resistive particles in a conductive solvent. Figure S2 shows the model results for the converse case when the particles are conductive and the solvent is resistive.

It is experimentally challenging to calculate χ as a function of time since resistivity requires a simultaneous transient measure of film resistance and total film thickness. However, it is useful to consider the ratio of the resistances for the smaller and larger spaced probe as a function of time, which does not require an independent measure of thickness. The ratio of the resistances is denoted by *α = R_s_/R*_*L*_, where the resistance observed by the smaller and larger spaced probes are denoted by *R*_*S*_ and *R*_*L*_, respectively. Figure 6 shows α for all three cases. In the diffusion regime, the only difference between the two observed resistances is caused by the shrinkage in film thickness due to the film drying homogeneously. Since the thickness is shrinking linearly, α also decreases linearly. Similarly to Figure 6b,c, the sedimentation and evaporation regimes are represented by an increase and a minimum in α respectively. The qualitative differences between the three functional forms suggest that the shape of the α curve can also be used to indicate the drying regime.

**Figure 7:**
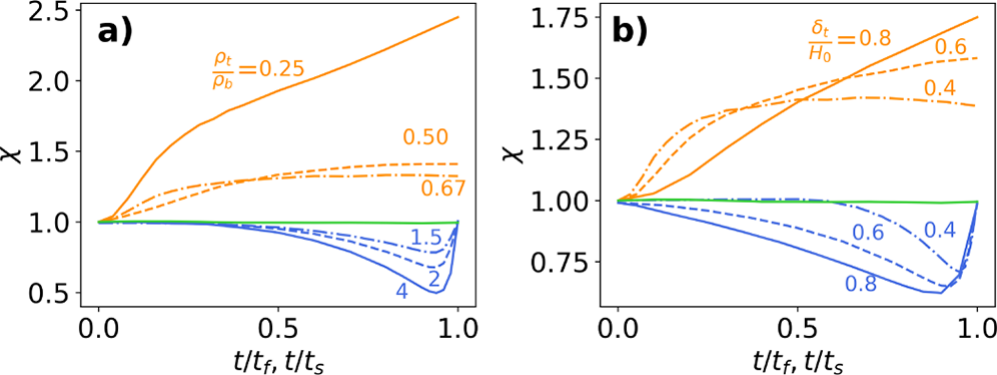
Changes in the three drying regimes represented in terms of χ for different values of (a) the final resistivity ratio between the two layers ( for evaporation,  for sedimentation) and (b) the normalized final top layer thickness ( for evaporation,  for sedimentation).

Except for the diffusion dominated case, the model results described in Figure 7 depend on the two normalized parameters ρ_*t*_/ρ_*b*_ and δ_*t*_/*H*_0_ evaluated at *t* > *t*_*f*_ or *t* > *t*_*s*_. Figure 7(a) shows the effect of changing the final resistivity ratio, ρ_*t*_/ρ_*b*_, on the three drying regimes. For the sedimentation regime, the magnitude of the increase in χ decreases with increasing ρ_*t*_/ρ_*b*_ and completely disappears when *ρ_t_/ρ*_*b*_ = 1. However, the functional form of χ does not change with the ratio of ρ_*t*_/ρ_*b*_. The opposite trend is observed for the evaporation regime, where the magnitude of the minimum decreases with decreasing ρ_*t*_/ρ_*b*_, but the functional form of χ stays the same. Thus, the ability to distinguish between the different drying regimes is strongly dependent on the differences in resistivity of the stratified layers. Figure 7b shows the effect of changing the final normalized top layer thickness, δ_*t*_/*H*_0_,on the three regimes. Unlike changing ρ_*t*_/ρ_*b*_, a change in δ_*t*_/*H*_0_ significantly alters the functional form of χ. For sedimentation, χ(*t*_*s*_) increases with increasing δ_*t*_/*H*_0_. Similarly for evaporation, the magnitude of the maximum in χ increases with increasing δ_*t*_/*H*_0_. These changes occur because the top layer is undetectable by the smaller spaced probe but detectable by the larger probe. Thus, as the top layer becomes larger, the discrepancy between the resistivities observed by the two probes increases. Therefore, the ability to distinguish between the different drying regimes is strongly dependent on the thickness of the top layer relative to the initial total film thickness.

**Figure 8:**
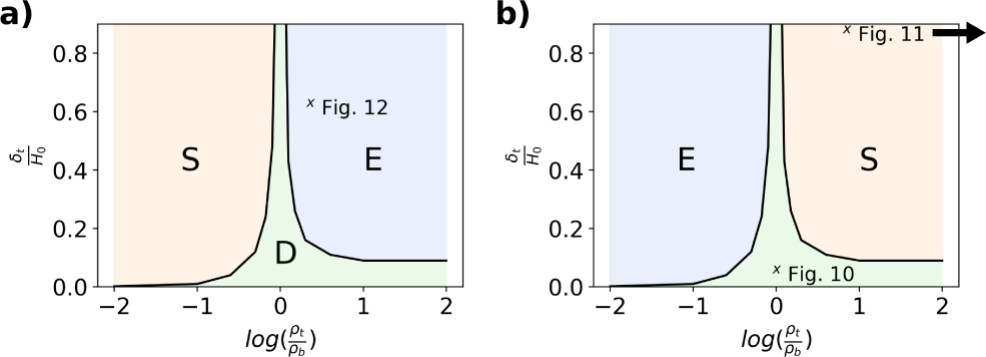
Maps for determining the feasibility of drying regime differentiation at different combinations of the final resistivity ratio and the final normalized thickness of the top layer. The two curves represent a statistically significant increase (left) and decrease (right) in χ. Map (a) is for resistive particles in conductive solvent, (b) is for conductive particles in resistive solvent. Each figure shows the predicted location of the proof-of-concept experiments assuming a maximum packing fraction of 0.64.

Figure 8a,b are processing maps considering the relative ratio of the top layer resistivity to the bottom layer resistivity, ρ_*t*_/ρ_*b*_, for different ratios of the top layer thickness normalized by the overall film thickness. The solid lines represent the boundaries of the different drying regimes and are defined by a minimum change in χ of 10% compared to unity. Figure 8a shows the map for resistive particles suspended in conductive solvent and Figure 8b, shows the opposite, i.e., conductive particles in resistive solvent. In Figure 8a, for small ratios of ρ_*t*_/ρ_*b*_, the top film thickness required to observe sedimentation is significantly reduced, and the measurement is very sensitive to stratification of the film. For large ratios of ρ_*t*_/ρ_*b*_, the top film thickness required to observe a 10% change in χ is much larger than sedimentation and relatively insensitive to the ratio of resistivity. When ρ_*t*_/ρ_*b*_ ≈ 1, the two curves diverge, indicating that neither sedimentation nor evaporation can be detected within the film. Figure 8b exhibits the same trends as (a) with the sedimentation and evaporation regions switched due to the inversion of ρ_*t*_/ρ_*b*_. These maps may be used to estimate a minimum value of  needed to distinguish the evaporation or the sedimentation regime from the diffusion regime. For example, if we estimate the maximum resistivity ratio of two distinct layers, one containing consolidated nonconducting particles with a maximum packing fraction of 0.64 (random close packing), and the other layer consisting of only a conductive solvent (i.e., bottom layer), then  for the evaporation regime, and  = 1/2.78 = 0.36 for the sedimentation regime. Figure 8 shows that a minimum of  and  is required to distinguish the evaporation and sedimentation regimes, respectively. The relative thickness of the top layer for both the sedimentation and evaporation dominated regimes were reported by Cardinal et al. for silica particles in water via Cryo-SEM imaging and are shown to be above this minimum requirement, suggesting that this methodology is capable of detecting the differences in sedimentation, diffusion, and evaporation dominated drying.^7^

#### Comparison of Heuristic and Physics-Based Drying Models

**Figure 9:**
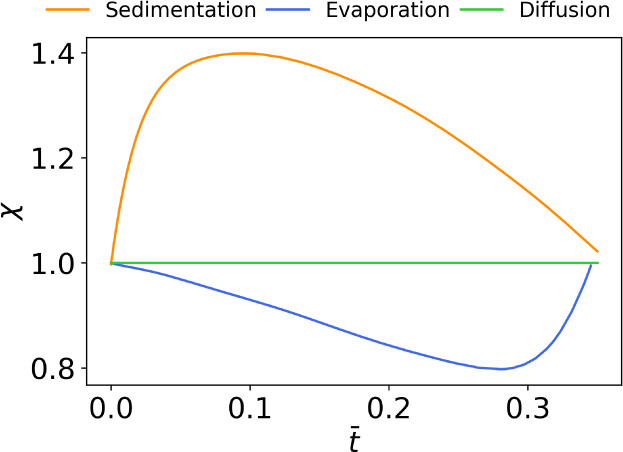
Three regimes represented in terms of χ when the heuristic colloidal drying model is replaced by a physics-based model found in literature. Pe = 1 and *N*_*s*_ = 1 for evaporation, Pe = 1 and *N*_*s*_ = 100 for the sedimentation. The normalized time variable is defined as .

Figure 9 shows the magnitude of χ for the four-point probe simulation using the vertical particle volume fraction profile determined from eqs 7–9. Here, the normalized time variable is defined as . The simulation ends when the film reaches its final thickness at *t̅* = 0.35. When compared to the results in Figure 6d, the evaporation regime in both models exhibits a minimum in χ at high *t* but the physics-based model shows a more linear decrease at low *t*. This discrepancy is caused by the heuristic model assumption that the consolidated skin forms as a discrete layer, while the physics-based model assumes a gradual change of dimensions. In the sedimentation regime, both models show an initial increase in χ but the physics-based model shows a subsequent decrease. This discrepancy is caused by the lack of solvent evaporation in the heuristic sedimentation model. As solvent evaporates, the thickness of the top layer approaches zero and χ approaches unity. Despite these differences, Figures 9 and 6 show qualitatively similar trends from the heuristic and physics-based models, thus validating the heuristic model used in this study.

### Experimental Results

The choice of solution and line-probe spacing met the constraints previously discussed in the Model Development and Numerical Results sections to ensure that a specific drying regime was observed. The experimental parameters are shown on the processing map in Figure 8. These parameters are estimated considering the initial solid content of the suspension and conductivity of the solvent. Details are given in the Table S1.

#### Diffusion Dominated Regime

**Figure 10:**
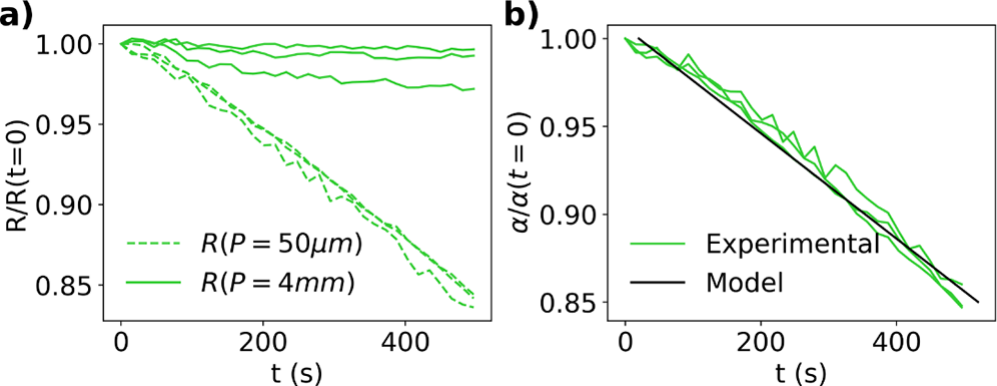
(a) Change in resistance measured by probes with *P* = 4 mm (solid line) and *P* = 50 μm (dashed line) when a droplet of an aqueous solution of 10 mM KCl dries on top of the line probes. (b) Change in α in the same experiment (green) with heuristic model result comparison (black) where *H*_*dry*_ = 0.85*H*_0_ and *t*_*f*_ = 5000 s. The additional lines represent repeat experiments.

Figure 10a shows the four-point probe result for a drying 10 mM KCl solution with *P* = 4 mm and *P* = 50 μm. The larger spaced probe measures a slight decrease in resistance, while the smaller spaced probe measures a large linear decrease in resistance with time. The negligible change measured by the larger spaced probe is due to the fact that the increase in resistance from decreasing film thickness is counteracted by the increasing ion concentration. However, the smaller spaced probe is incapable of detecting the air–liquid interface and therefore only senses the linearly increasing ion concentration. Figure 10b shows that the ratio of the two probes’ resistances, α = *R*(*P* = 50 μm)/*R*(*P* = 4 mm) decreases linearly with time, which agrees well with the trend in Figure 6e. Additionally, the film thickness and thus the evaporation rate may be approximated using the relation , where *S*(*small*) is the shape factor for the smaller spaced probe and *L* is the length of the line probe in contact with the sample. Here, *E* was calculated to be 1.32 ± 0.02 μm s^–1^. This value is within the same order of magnitude of the interface velocity measured using a digital displacement micrometer of colloidal solutions, *E* = 0.47 ± 0.05 μm s^–1^, see Figure S1 for additional details.

#### Sedimentation Dominated Regime

**Figure 11:**
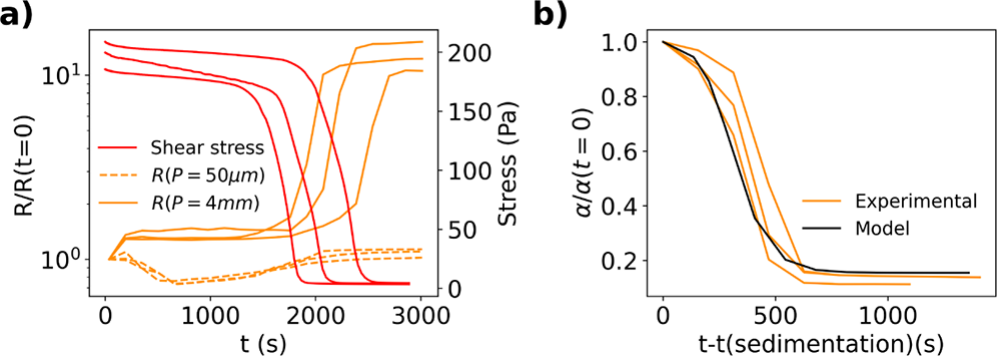
(a) Primary *y*-axis: Change in resistance measured by probes with *P* = 4 mm (orange solid line) and *P* = 50 μm (orange dashed line) while the carbon black slurry is under shear. Secondary *y*-axis: Stress response as the carbon black slurry (red solid line) is being sheared at 100 s^–1^ while on top of the four-line probe. The stars, squares and circles are used to match the shear stress curve to the corresponding resistance measurement. *t*(*sedimentation*) for the experiments marked in stars, squares and circles are taken as 2071, 1445, and 1758 s, respectively. (b) Change in α in the same experiment including heuristic model comparison where ρ_*t*_/ρ_*b*_ = 2856, δ_t_/*H*_0_ = 0.996 and *t*_*s*_ = 1360 s. The additional lines represent repeat experiments.

Figure 11(a) shows the stress response measured by a parallel plate rheometer for three samples of carbon black and propylene carbonate slurry sheared at 100 s^–1^. This experimental condition mimics the case when drying is much slower than sedimentation. The bottom plate is a four-line probe device. The measurement exhibits an initial slight decrease in shear stress followed by a sharp decrease at *t* ∼2000 s, which denotes the onset of slurry sedimentation.^19^ When sedimentation occurs, the conductive carbon black particles fall to the bottom, leaving a resistive solvent supernatant that can only be detected by the larger probe. Figure 11a also shows the resistance measured by the line probes with *P* = 4 mm and 50 μm while the carbon black slurry is sheared at 100 s^–1^. The resistance of the larger probe increases sharply at the onset of sedimentation. Although there is variation in time of sedimentation for all three samples, there is excellent agreement between the time of sedimentation measured from stress and the increase in resistance. The variation between repeat experiments in this data set is attributed to sample thixotropy. The time taken to load the sample, start the experiment and the method of loading impact the slurry history and thus the dynamics of structural evolution, i.e., the resistance versus time.^31^ A preshear protocol was used to reduce the magnitude of these effects but could not completely eliminate them. Interestingly, despite the increased concentration of conductive carbon near the electrodes, the smaller spaced probe does not measure a significant decrease in resistance. Although at early times before sedimentation we observe a slight decrease in resistance, the resistance at later times increases back to the initial value. One explanation is that the decreased resistance from higher local carbon concentration is counteracted by an increased resistance due to the loss of carbon connectivity during shear. It is not clear what triggers the loss of connectivity at intermediate times. Figure 11b summarizes the resistance results via α after the time of sedimentation (*t*(*sedimentation*)). Note that the subtraction of the sedimentation shows that the different experiments have very similar sedimentation rates. Changes in α before this time is beyond the scope of this work. While Figure 6e models the case of resistive particles in conductive solvent, the line in Figure 11b represents the case of conductive particles in resistive solvent, as in the case of Figure S2b. Note that the parameters for the model were determined using experimentally relevant time scales and ratios of conductivities. The model shows excellent qualitative agreement with the experimental data suggesting that no additional physics are required to explain the sedimentation dynamics of resistance with time.

#### Evaporation Dominated Regime

**Figure 12:**
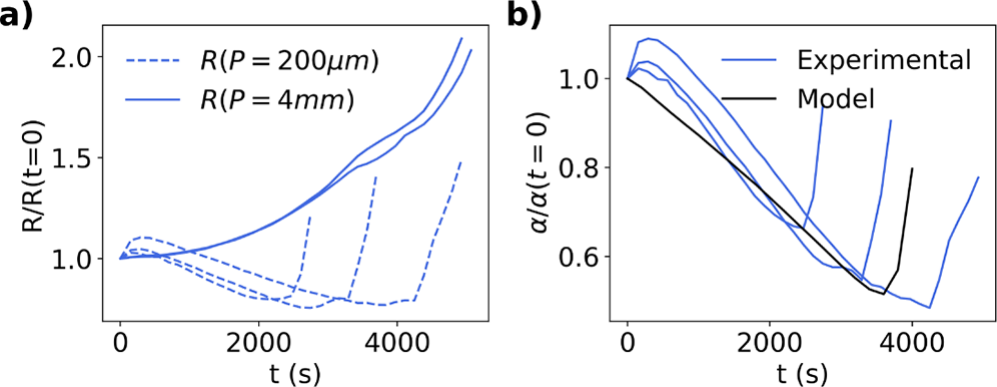
(a) Change in resistance measured by probes with *P* = 4 mm (solid line) and *P* = 200 μm (dashed line) silica suspension is drying. (b) Change in α in the same experiment including heuristic model comparison where ρ_*t*_/ρ_*b*_ = 22, δ_*t*_/*H*_0_ = 0.8 and *t*_*f*_ = 500 s. The additional lines represent repeat experiments.

Figure 12a shows the change in resistance measured by the line probes with *P* = 4 mm and 200 μm for an aqueous suspension of silica particles in 10 mM KCl. The film dries at room temperature under forced convection with Pe = 77.7 and *N*_*s*_= 2 × 10^–4^. The larger probe measures an increase in resistance throughout the drying process as the concentration of resistive silica particles increases near the air–liquid interface. For the most part, the smaller spaced probe measures a decrease in resistance followed by an increase. Note that we do not have a reason for the slight increase in resistance at very early times. The initial decrease in resistance for the smaller spaced probe is due to the local increasing salt concentration from solvent evaporation and osmotic pressure forcing water molecules into the interstitial sites of consolidated particles. The increased resistance at later times occurs when the film is nearly dry, and the smaller spaced probe detects the consolidated particle layer. Figure 12b shows the change in α during drying where α = *R*(*P* = 200 μm)/*R*(*P* = 4 mm), which agrees well with heuristic model trend in Figure 6e. The solid black line in Figure 12b shows the model trend for characteristic parameters.

Note that it is very difficult to control evaporation rates in thin film drying.^32^ Variation between repeat experiments is attributed to changes in evaporation rate caused by slight variations in sample area, ambient humidity, local convection rates, and contact angle (shape of the film). Although there are differences in evaporation rate, the experimental films have very similar trends in resistivity.

## Conclusions

This study showed that multiple four-point probes with different spacings are capable of determining microstructural changes in drying films using the principles of geophysical prospecting. Model simulations predict that differences between the resistivity measured by two sensors can distinguish between diffusion, sedimentation, and evaporation dominated drying. In order to distinguish drying regimes, the larger spaced electrode spacing must exceed twice the initial film thickness, and the spacing of the smaller spaced probe cannot exceed half the expected thickness of the bottom stratified layer. These two constraints ensure a uniform electric field and that the electric field only penetrates into the bottom layer, respectively. Note that the constraints are for idealized equidistant electrodes of infinitesimal width and thickness, and the critical spacing for electrodes of finite thickness or width may vary. For example, the smaller spaced experimental probes used in this study differed from the theoretical limit by a factor of 2.5.

The theoretical predictions were validated via qualitative agreement with experimental trends. The results clearly show that the three drying regimes of diffusion, sedimentation, and evaporation have distinct trends in resistivity and resistance. The trends in resistance, i.e., *α*, are easier to measure and do not require independent measurements of film thickness. Diffusion dominated drying shows a linear trend in *α* that is directly proportional to the interface velocity. Considering insulated particles in a conductive solvent, sedimentation dominated drying shows a nonlinear increasing function of α as the particles settle near the electrode substrate. Lastly, evaporation dominated drying shows a distinct minimum in α that corresponds to the consolidated front at the top of the film as a function of time. Overall, the method developed in this work clearly differentiates between the three drying mechanisms and, since the final microstructure is determined by the mechanism, will aid in better understanding processing-structure–function relationships in a range of applications. For example, the probes could be used to investigate the dynamics of binder migration in lithium ion battery electrodes,^6^ or the irregular influence of shear rate on electrode performance.^1^

An obvious limitation of this method is that either the colloidal film or the solvent must be electronically or ionically conductive. This can be overcome by either adding a small amount of salt to the solvent or applying a very high voltage. However, both of these solutions may influence the microstructure, e.g., adding salt may increase particle attraction, and applying high voltage may induce electrochemical reactions or electrophoresis. Another limitation is that the method requires an electode substrate be deposited on the substrate of interest, which may limit its applicability to certain substrates and geometries. This method may be improved by adding more sensors of different spacings to obtain a more complete understanding of the vertical resistivity profile. Despite these limitations, the results of this work present a low-cost, experimentally accessible method for investigating drying mechanisms across many different systems and applications.

## Supporting Information Available

Includes experimental results of the evaporation rate measurement, the three regimes represented in terms of χ and α when the particles are conductive and the solvent is resistive, the impact of the stratified layer discreteness (*d*) on the two observed resistivities *ρ*_*s*_ and *ρ*_*L*_ and details on how *ρ*_*t*_/*ρ*_*b*_ and δ_*t*_/*H*_0_ were approximated for the three proof-of-concept experiments (PDF)

## Nomenclature

*d*: Constant determining the discreteness of the two stratified layers
*E*: Evaporation rate in terms of interface velocity, mm/s
*H*(*t*): Transient film thickness, mm
*H*_0_: Initial film thickness, mm
*H*_*dry*_: Dry film thickness, mm
*H*_*sed*_: Final sediment layer thickness, mm
*K*: *ρ*_*b*_(*t*)/*ρ*_*t*_(*t*)
*P*: Spacing between adjacent electrodes, mm
*p*: Constant of proportionality
*R*: Resistance, Ω
*R*_*L*_: Resistance observed by large spaced probe, Ω
*R*_*S*_: Resistance observed by small spaced probe, Ω
*t*: Time, s
*t*_*f*_: Final drying time, s
*t*_*S*_: Final sedimentation time, s
*t*(*sedimentation*): Experimental time of sedimentation, s
*Et*/*H*_0_
*U*: Sedimentation velocity, mm/s
*V*_*S*_: Sediment Layer growth velocity, mm/s
*y*: 2D vertical spatial coordinate, mm
*z*: 3D vertical spatial coordinate, mm
α: *R*_*S*_/*R*_*L*_
δ_*b*_: Final bottom layer thickness, mm
δ_b_(t): Transient bottom layer thickness, mm
δ_b_(max): Maximum bottom layer thickness, mm
δ_*t*_: Final top layer thickness, mm
δ_t_(t): Transient top layer thickness, mm
ζ: *z*/*H*_0_
ρ: Resistivity of the film, Ω mm
ρ_0_: Initial resistivity of the film, Ω mm
ρ(t): Transient resistivity of the film, Ω mm
ρ(z, t): Resistivity of the film as a function of the vertical coordinate and time, Ω mm
ρ_*b*_: Final resistivity of the bottom layer, Ω mm
ρ_b_(t): Transient resistivity of the bottom layer, Ω mm
ρ_*t*_: Final resistivity of the top layer, Ω mm
ρ_t_(t): Transient resistivity of the top layer, Ω mm
ρ_*L*_: Resistivity observed by large spaced probe, Ω mm
ρ_*S*_: Resistivity observed by small spaced probe, Ω mm
ρ_*f*_: Final resistivity of the dried film, Ω mm
ρ_f,sup_: Final resistivity of the supernatant, Ω mm
τ_*E*_: Evaporation time constant, s
τ_*S*_: Sedimentation time constant, s
ϕ: Electric potential, V
Φ: Particle volume fraction
χ: ρ_*S*_ /ρ_*L*_
ψ: δ_*b*_(*t*)/*H*(*t*)

## References

[ref1] Saraka R. M.; Morelly S. L.; Tang M. H.; Alvarez N. J. Correlating processing conditions to short- and long-range order in coating and drying lithium-ion batteries. ACS Appl. Energy Mater. 2020, 3, 11681–11689. 10.1021/acsaem.0c01305.

[ref2] Tiarks F.; Frechen T.; Kirsch S.; Leuninger J.; Melan M.; Pfau A.; Richter F.; Schuler B.; Zhao C. L. Effects on the pigment distribution in paint formulations. Macromol. Symp. 2002, 187, 739–752. 10.1002/1521-3900(200209)187:1<739::AID-MASY739>3.0.CO;2-M.

[ref3] Yari S.; Hamed H.; D’Haen J.; Bael M. K. V.; Renner F. U.; Hardy A.; Safari M. Constructive versus destructive heterogeneity in porous electrodes of lithium-ion batteries. ACS Appl. Energy Mater. 2020, 3, 11820–11829. 10.1021/acsaem.0c01966.

[ref4] Dominko R.; Gaberscek M.; Drofenik J.; Bele M.; Pejovnik S.; Jamnik J. The role of carbon black distribution in cathodes for li ion batteries. J. Power Sources 2003, 119–121, 770–773. 10.1016/S0378-7753(03)00250-7.

[ref5] Bauer W.; Nötzel D.; Wenzel V.; Nirschl H. Influence of dry mixing and distribution of conductive additives in cathodes for lithium ion batteries. J. Power Sources 2015, 288, 359–367. 10.1016/j.jpowsour.2015.04.081.

[ref6] Jaiser S.; Müller M.; Baunach M.; Bauer W.; Scharfer P.; Schabel W. Investigation of film solidification and binder migration during drying of li-ion battery anodes. J. Power Sources 2016, 318, 210–219. 10.1016/j.jpowsour.2016.04.018.

[ref7] Cardinal C. M.; Jung Y. D.; Ahn K. H.; Francis L. F. Drying regime maps for particulate coatings. AichE J. 2010, 56, 2769–2780. 10.1002/aic.12190.

[ref8] Kameya Y. Kinetic monte carlo simulation of nanoparticle film formation via nanocolloid drying. J. Nanopart. Res. 2017, 19, 108002 10.1007/s11051-017-3898-3.

[ref9] Zhou J.; Jiang Y.; Doi M. Cross interaction drives stratification in drying film of binary colloidal mixtures. Phys. Rev. Lett. 2017, 118, 108002 10.1103/PhysRevLett.118.108002. 28339235

[ref10] Luo H.; Cardinal C. M.; Scriven L. E.; Francis L. F. Ceramic nanoparticle/monodisperse latex coatings. Langmuir 2008, 24, 5552–5561. 10.1021/la800050u. 18416565

[ref11] Luo H.; Scriven L. E.; Francis L. F. Cryo-sem studies of latex/ceramic nanoparticle coating microstructure development. J. Colloid Interface Sci. 2007, 316, 500–509. 10.1016/j.jcis.2007.07.047. 17854820

[ref12] Prakash S. S.; Francis L. F.; Scriven L. E. Microstructure evolution in dry cast cellulose acetate membranes by cryo-sem. J. Membr. Sci. 2006, 283, 328–338. 10.1016/j.memsci.2006.07.001.

[ref13] Cardinal C. M.; Francis L. F.; Scriven L. E. Drying and collapse of hollow latex. J. Coat. Technol. Res. 2009, 6, 457–469. 10.1007/s11998-009-9167-3.

[ref14] Buss F.; Roberts C. C.; Crawford K. S.; Peters K.; Francis L. F. Effect of soluble polymer binder on particle distribution in a drying particulate coating. J. Colloid Interface Sci. 2011, 359, 112–120. 10.1016/j.jcis.2011.03.054. 21497825

[ref15] Ma M.; Jiang K.; Qiu G.; Wang D.; Hu X.; Jin X.; Chen Z. G. Fundamental study on electro-reduction of solid titania in molten calcium chloride. J. Rare Earths 2005, 23, 46–49.

[ref16] Li J.; Cabane B.; Sztucki M.; Gummel J.; Goehring L. Drying dip-coated colloidal films. Langmuir 2012, 28, 200–208. 10.1021/la203549g. 22053849

[ref17] Dingenouts N.; Ballauff M. First stage of film formation by latexes investigated by small-angle x-ray scattering. Langmuir 1999, 15, 3283–3288. 10.1021/la9816510.

[ref18] Dingenouts N.; Ballauff M. Assessment of spatial order in dried latexes by small-angle x-ray scattering. Macromolecules 1998, 31, 7423–7429. 10.1021/ma980682z.

[ref19] Hipp J. B.; Richards J. J.; Wagner N. J. Structure-property relationships of sheared carbon black suspensions determined by simultaneous rheological and neutron scattering measurements. J. Rheol. 2019, 63, 423–436. 10.1122/1.5071470.

[ref20] Ekanayake P.; Mcdonald P. J.; Keddie J. L. An experimental test of the scaling prediction for the spatial distribution of water during the drying of colloidal films. EPJ: Spec. Top. 2009, 166, 21–27. 10.1140/epjst/e2009-00872-4.

[ref21] Salamanca J. M.; Ciampi E.; Faux D. A.; Glover P. M.; McDonald P. J.; Routh A. F.; Peters A. C. I. A.; Satguru R.; Keddie J. L. Lateral drying in thick films of waterborne colloidal particles. Langmuir 2001, 17, 3202–3207. 10.1021/la001590h.

[ref22] Wallin M.; Glover P. M.; Hellgren A. C.; Keddie J. L.; McDonald P. J. Depth profiles of polymer mobility during the film formation of a latex dispersion undergoing photoinitiated cross-linking. Macromolecules 2000, 33, 8443–8452. 10.1021/ma000787d.

[ref23] Narayanan A.; Mugele F.; Duits M. H. G. Mechanical history dependence in carbon black suspensions for flow batteries: A rheo-impedance study. Langmuir 2017, 33, 1629–1638. 10.1021/acs.langmuir.6b04322. 28122184 PMC5333906

[ref24] Liu Q.; Richards J. J. Rheo-electric measurements of carbon black suspensions containing polyvinylidene difluoride in n -methyl-2-pyrrolidone. J. Rheol. 2023, 67, 647–659. 10.1122/8.0000615.

[ref25] Bai S. J.; Song Y. S. Correlation between internal structure and electrochemical impedance spectroscopy of multiphase slurry systems. Anal. Chem. 2013, 85, 3918–3925. 10.1021/ac303187r. 23480271

[ref26] George F. V. K.; Frischknecht C. Electrical Methods in Geophysical Prospecting; Ergaon Press Inc.: Oxford, 1966; Vol. 66, pp. 100–177.

[ref27] Lanterman B. J.; Riet A. A.; Gates N. S.; Flygare J. D.; Cutler A. D.; Vogel J. E.; Wheeler D. R.; Mazzeo B. A. Micro-four-line probe to measure electronic conductivity and contact resistance of thin-film battery electrodes. J. Electrochem. Soc. 2015, 162, A2145–A2151. 10.1149/2.0581510jes.

[ref28] Shiraki I.; Tanabe F.; Hobara R.; Nagao T.; Hasegawa S. Independently driven four-tip probes for conductivity measurements in ultrahigh vacuum. Surf. Sci. 2001, 493, 633–643. 10.1016/S0039-6028(01)01276-6.

[ref29] Clarysse T.; Eyben P.; Parmentier B.; Daele B. V.; Satta A.; Vandervorst W.; Lin R.; Petersen D. H.; Nielsen P. Advanced carrier depth profiling on si and ge with micro four-point probe. Vac. Sci. Technol. B 2008, 26, 317–321. 10.1116/1.2802101.

[ref30] Newman J.; Balsara N. P. Electrochemical Systems, 4th ed.; Wiley: Hoboken, N.J, 2020; pp. 449–488.

[ref31] Mewis J.; Wagner N. J. Thixotropy. Adv. Colloid Interface Sci. 2009, 147–148, 214–227. 10.1016/j.cis.2008.09.005. 19012872

[ref32] Ternes S.; Börnhorst T.; Schwenzer J. A.; Hossain I. M.; Abzieher T.; Mehlmann W.; Lemmer U.; Scharfer P.; Schabel W.; Richards B. S.; et al. Drying dynamics of solution-processed perovskite thin-film photovoltaics: In situ characterization, modeling, and process control. Adv. Energy Mater. 2019, 9, 2490 10.1002/aenm.201901581.

