# Supplementary material for: Microscale Electrical Resistivity Measurements to Investigate Particle Distribution
Source: Langmuir. 2025 Jan 7;41(2):1231–40. doi: 10.1021/acs.langmuir.4c03429 (PMC11755783; doi:10.1021/acs.langmuir.4c03429)
Supplement: Supplementary file 1 — la4c03429_si_001.pdf [file la4c03429_si_001.pdf]

Supporting Information

Emre Baburoglu[1], Maureen H. Tang[1,2], Nicolas J. Alvarez[1,2*]

[1*]Materials Science and Engineering, Drexel University, 3141 Chestnut Street, Philadelphia, 19104, PA, USA.
[2*]Chemical and Biological Engineering, Drexel University, 3141 Chestnut Street, Philadelphia, 19104, PA, USA.

*Corresponding author(s). E-mail(s): alvarez@drexel.edu;
Contributing authors: eb937@drexel.edu; mhtang@drexel.edu;

Number of pages: 5
Number of figures: 4
Number of tables: 1

Contents

1 Evaporation rate measurements **S2**

2 Case where the particles and conductive and the solvent is resistive **S2**

3 Impact of Stratified Layer Discreteness **S3**

4 ρ_t/ρ_b and δ_t/H_0 approximations **S5**

1 Evaporation rate measurements

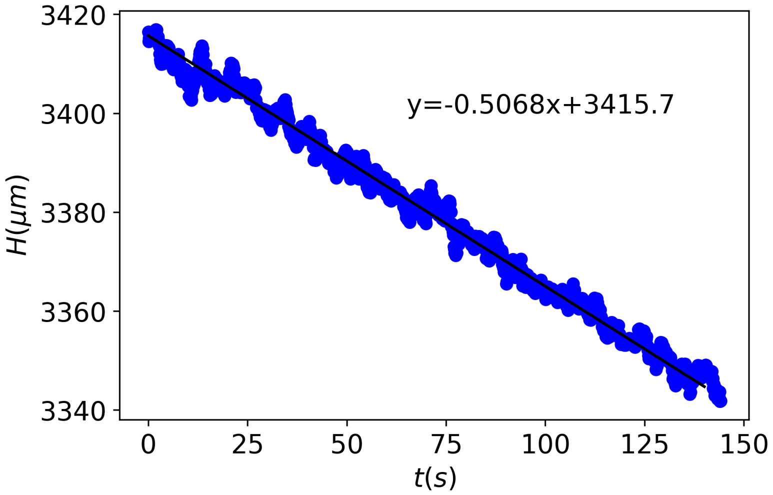

Figure S 1 Change in thickness of a suspension consisting of 0.4 volume fraction silica in an aqueous solution of 10 mM KCl under forced convection (blue). The fitted trend line and equation is shown in black. The thickness measurement is performed by a digital displacement micrometer.

Figure S1 shows the results of the thickness measurement performed by a digital displacement micrometer while a suspension consisting of 0.4 volume fraction silica in an aqueous solution of 10 mM KCl is being dried under forced convection. Here, 700 μL of the solution is dispensed onto an 18 x 18 mm area of the four-line probe device. The slope of the fitted trend line shown in black is the evaporation rate in terms of the velocity of the interface. The average evaporation rate over three repeats was 0.47±0.05 μm s^{-1}.

2 Case where the particles and conductive and the solvent is resistive

Figure S2 shows the three regimes represented in terms of χ and α for conductive particles suspended in insulating solvent. As expected, the trends depicting the sedimentation and evaporation regimes are reversed when compared to Figure 5, with the characteristic increase in the sedimentation regime becoming a decrease, and the characteristic minimum in the evaporation regime becoming a maximum. The trends depicting the diffusion regime, however, remain the same because dividing the probe resistances eliminates the impact of uniform changes in resistivity on α and χ. Thus, the functional form of the trends in α and χ for diffusion-dominated drying is independent of whether the solvent or the particles are electrically conductive.

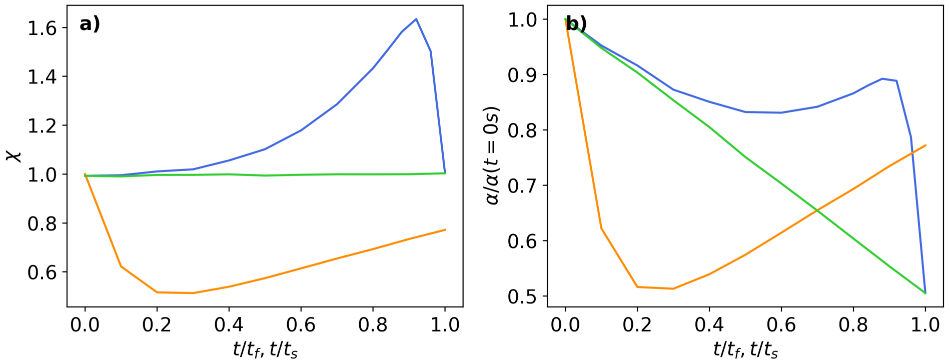

Figure S 2 The three regimes represented in terms of a)χ and b)α when the particles are conductive and the solvent is insulating. Here, $\frac{\rho_t}{\rho_b} = 2$ and $\frac{\delta_t}{H_0} = 0.5$ for sedimentation and $\frac{\rho_t}{\rho_b} = 0.5$, $\frac{\delta_t}{H_0} = 0.5)$ for evaporation.

3 Impact of Stratified Layer Discreteness

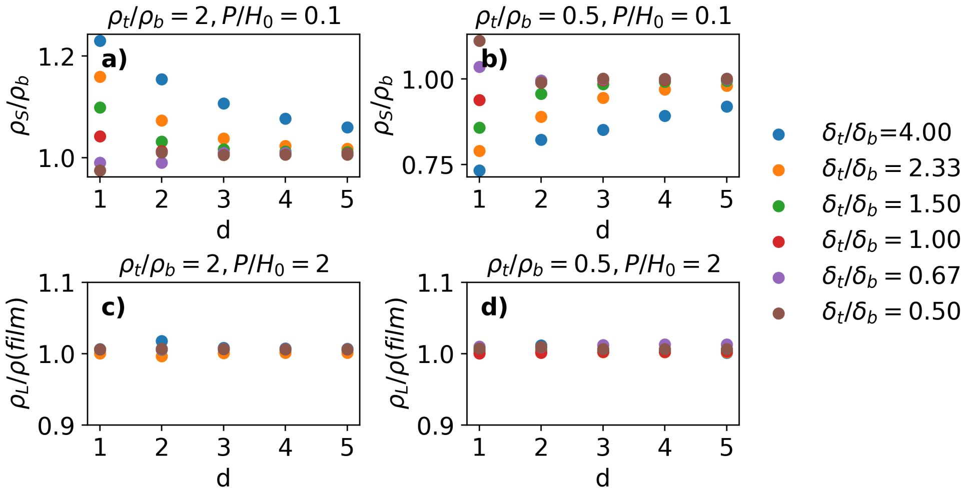

Figure S 3 Plots depicting the impact of the discreteness of the two stratified layers on the resistivity observed by the two probes depending on their relative resistivities and layer thicknesses. Where a) $\rho_t/\rho_b = 2$ and P/H_0=0.1, b) $\rho_t/\rho_b = 0.5$ and P/H_0=0.1, c) $\rho_t/\rho_b = 2$ and P/H_0=2, d)$\rho_t/\rho_b = 0.5$ and P/H_0=2.

Figure S3(a) depicts the impact of changing the parameter d on the resistivity observed by the small spaced probe (ρ_S) for when the top of the film is twice as resistive as the bottom for different values of the ratio of the layer thicknesses, or δ_t/δ_b. Figure

S3(b) shows the results of the same simulation for when the bottom of the film is twice as resistive as the top. Here, increasing d increases the sharpness of the interface between the two layers. In both cases, ρ_S approaches ρ_b as d increases for all values of δ_t/δ_b. This is due to the top layer-bottom layer interface moving further away from the probe, making the top layer less detectable. For the same reason, as δ_t/δ_b increases, ρ_S moves further away from ρ_b. When d >2 and $\delta_t/\delta_b < 4$ (i.e. $\delta_t/H_0 > 0.2$), ρ_S does not depend strongly on d for all other values of δ_t/δ_b. Figure S3(c) and (d) depict the impact of changing the parameter d on the resistivity observed by the large spaced probe (ρ_L) relative to the actual resistivity of the film ($\rho(film)$). Figure S3(c) depicts the case where the top of the film is twice as resistive as the bottom while Figure (d) depicts the opposite case. Both cases are examined for different values of δ_t/δ_b. From these results, it can be seen that ρ_L is independent of d for all δ_t/δ_b.

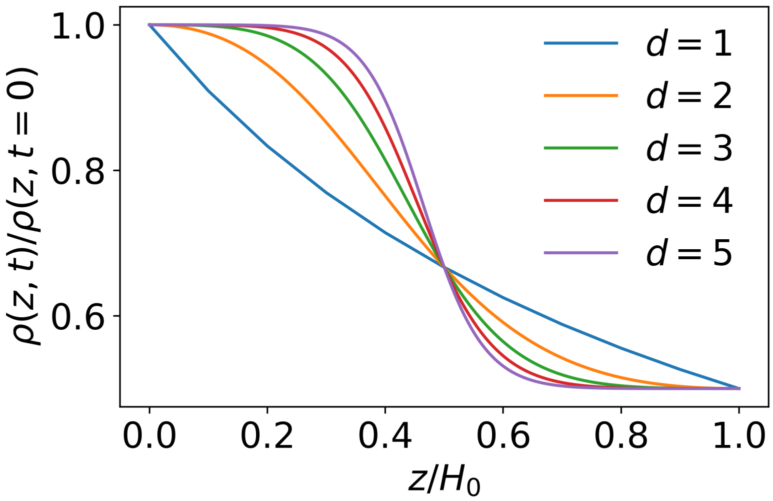

Figure S 4 Plot depicting the change in slope of the top layer-bottom layer interface with d. Here, δ_b/H_0=0.5 and δ_t/δ_b=2.

Figure S4 qualitatively depicts the impact of the parameter d on the sharpness of the change in resistivity from the bottom to the top of the film. When $d = 1$, resistivity changes gradually. As d increases, an inflection point forms and resistivity changes more drastically at the inflection point.

4 ρ_t/ρ_b and δ_t/H_0 approximations

Table S 1: Table depicting the approximations made for ρ_t/ρ_b and δ_t/H_0 for the three proof of concept experiments.

Regime	Suspension	ρ_t/ρ_b	δ_t/H_0
Diffusion	10 mM KCl	1	N/A
Sedimentation	8 wt% carbon black in propylene carbonate	>>100	0.925
Evaporation	0.4 volume fraction SiO_2 in 10 mM KCl	1.58	0.625

Table S1 shows the approximations made for ρ_t/ρ_b and δ_t/H_0 for the three proof-of-concept experiments. When an aqueous solution of 10 mM KCl dries, ρ_t/ρ_b must be equal to 1 as ionic solutions dry uniformly. For the sedimentation of carbon black, the resistivity ratio between the two layers is approximated as large since the supernatant is assumed to be purely propylene carbonate which is insulating. The normalized thickness of the top layer was approximated by converting the weight fraction to volume fraction and assuming random close packing for the packing fraction of the sediment. A 0.08 weight fraction in this case equates to 0.048 volume fraction. Thus, the maximum normalized thickness of the sediment must be 0.048/0.64=0.075 and that of the supernatant, or δ_t/H_0, 1-0.075=0.925. The resistivity ratio for the evaporation dominated drying experiment was approximated by assuming random close packing for the consolidated top layer of particles and the initial volume fraction for the bottom layer. Thus, the volume fraction of the conductive solution is 0.36 and 0.6 for the top and bottom respectively, which equates to a resistivity ratio of 1.58. The normalized top layer thickness was calculated in a similar way to the sedimentation case where δ_t/H_0=0.4/0.64=0.625.
